# A Novel Photoplethysmography Sensor for Vital Signs Monitoring from the Human Trachea

**DOI:** 10.3390/bios9040119

**Published:** 2019-10-02

**Authors:** James M. May, Justin P. Phillips, Tracey Fitchat, Shankar Ramaswamy, Saowarat Snidvongs, Panayiotis A. Kyriacou

**Affiliations:** 1 Research Centre for Biomedical Engineering, City, University of London, London EC1V 0HB, UK; Justin.Phillips.1@city.ac.uk (J.P.P.); p.kyriacou@city.ac.uk (P.A.K.); 2 Barts and the London NHS Trust, London E1 1BB, UK; Tracey.Fitchat@bartshealth.nhs.uk (T.F.); Shankar.Ramaswamy@bartshealth.nhs.uk (S.R.); Saowarat.Snidvongs@nhs.net (S.S.)

**Keywords:** photoplethysmography, sensing endotracheal tube, pulse oximetry, vital signs monitoring

## Abstract

Current pulse oximeter sensors can be challenged in working accurately and continuously in situations of reduced periphery perfusion, especially among anaesthetised patients. A novel tracheal photoplethysmography (PPG) sensor has been developed in an effort to address the limitations of current pulse oximeters. The sensor has been designed to estimate oxygen saturation (SpO_2_) and pulse rate, and has been manufactured on a flexible printed circuit board (PCB) that can adhere to a standard endotracheal (ET) tube. A pilot clinical trial was carried out as a feasibility study on 10 anaesthetised patients. Good quality PPGs from the trachea were acquired at red and infrared wavelengths in all patients. The mean SpO_2_ reading for the ET tube was 97.1% (SD 1.0%) vs. the clinical monitor at 98.7% (SD 0.7%). The mean pulse rate for the ET sensor was 65.4 bpm (SD 10.0 bpm) vs. the clinical monitor at 64.7 bpm (SD 9.9 bpm). This study supports the hypothesis that the human trachea could be a suitable monitoring site of SpO_2_ and other physiological parameters, at times where the periphery circulation might be compromised.

## 1. Introduction

Pulse oximetry is perhaps the most important development in patient monitoring in the last forty years, however its limitations are well known and well documented [1,2,3,4]. Despite improvements in the accuracy of pulse oximetry brought about by advances in signal processing over the last decade or so [5], reliable and dependable SpO_2_ readings still depend on adequate periphery perfusion, especially in anaesthetised patients

While seldom reported in literature these instances do happen [6]. There is a recognised need for improved pulse oximetry performance in some groups of vulnerable patients, especially as current coping methods only compromise patient safety and place an unwanted burden on the surgical team as they try to restore monitoring.

To overcome this limitation, Kyriacou et al. described an internally placed reflectance pulse oximetry system in 2001 that allowed detailed investigation of SpO_2_ measurements and the photoplethysmography (PPG) signals, from which the SpO_2_ signals are derived from the oesophageal wall [7]. The oesophagus was chosen as a potentially suitable central monitoring site as it is readily accessible in most anaesthetised patients and is perfused directly by main arteries.

An initial clinical study, performed in patients undergoing general anaesthesia showed that reliable oesophageal signals could be obtained in all subjects, and that the optical pulse signals were significantly larger in amplitude than those obtained from a probe placed on the finger. A follow-on clinical study [8,9] investigated and compared oesophageal and finger PPG signals and SpO_2_ measurements in 49 patients undergoing hypothermic cardiothoracic bypass surgery. Measurable PPG traces at red and infrared wavelengths were obtained in the oesophagus in all 49 patients. Of the 49 patients included in the study, it was found that five patients had one or more periods of at least ten consecutive minutes during which the commercial finger pulse oximeter failed to display SpO_2_ values, despite being correctly positioned on the finger. Conversely, the oesophageal pulse oximeter operated successfully throughout these periods.

Internal sensors could offer significant improvements in accuracy and reliability over periphery sensors in cases of compromised periphery perfusion. In addition to the oesophagus the wall of the trachea is so far an unexplored but readily accessible and highly vascularised tissue bed and offers a single central site from which a wide range of clinical variables may be measured. There is also the additional advantage that every patient is intubated and sensors could be built directly into the endotracheal (ET) tube, thus eliminating the need for additional sensors to be placed such as was the case with the oesophagus. This work proposes a new novel and flexible tracheal PPG sensor. The design of the sensor and preliminary findings on five patients have been previously reported [10]. Initial findings show pulse oximetry and HR readings were in broad agreement. The work presented here describes the development and continuing clinical evaluation of the endotracheal PPG sensor.

## 2. Materials and Methods

### 2.1. The Endotracheal Sensor

**Figure 1:**
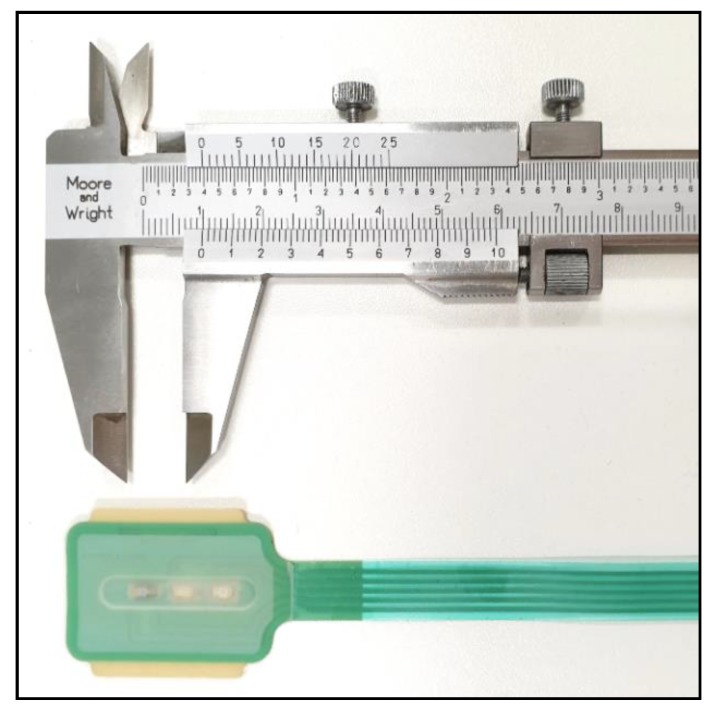
The endotracheal photoplethysmography (PPG) sensor before installation onto the endotracheal (ET) tube.

Work previously carried out on designing and constructing flexible reflectance PPG sensors with unusual geometry [11,12] was adapted to produce a sensor fit for the purpose of placing in the human trachea. A custom, flexible, printed circuit board (PCB) and silicone rubber covering were modelled in SolidWorks (Dassault Systèmes, Vélizy-Villacoublay, France). Due to the requirement of the sensor having to be attached to current standard endo-tracheal tubes (adult sizes 7 and 8), a compromise was made on optical component placement and geometry, most notably the components had to be arranged linearly, with the red LED closer to the photo diode. The centre-to-centre distances of the red and infrared LED to the photodiode were 7 mm and 13 mm, respectively. Normal reflectance sensors utilise a single dual LED package consisting of the 2 wavelengths of interest so that the LEDs are equidistant from the detector and usually no further than 5 mm. A photograph of the sensor before installation onto the ET tube is shown in Figure 1. Overall dimensions of the sensor, including the silicone covering are 435 × 29 × 1.3 mm (L × W × H).

The PCB carrying the PPG probe was manufactured on a polyester film with a thickness of 75 µm (Melinex, DuPont, Midland, MI, USA) with a sensor interface at the distal end incorporating one 660 nm (red) and one 940 nm (infrared) LED (KP-3216 series LEDs, Kingbright, Taipei, Taiwan) and a broad spectrum (400–1100 nm) high sensitivity PIN photo diode (VEMD6010 × 01, Vishay Semiconductors, PA, USA). The components were bonded to the PCB with a flexible epoxy resin (Light Weld 488-Series, DYMAX, USA) and electrically and thermally insulated with the custom 3D-modelled, clear, silicone rubber covering (Silpuran 6000, Wacker Chemie, Munich, Germany). The circuit board was insulated with UV-cured flexible ink (Electrodag 452SS, Henkel, Düsseldorf, Germany) and terminated at the proximal end by a 5-pin connector (Crimpfelx, Nicomatic, France).

The proposed sensor was designed to be single use, hence following the successful completion and technical evaluation of a single prototype sensor a batch of 50 sensors was developed for use in the pilot clinical trial. All probes were cleaned, sealed in blister packs and chemically sterilised (ETO procedure) by a specialised sterilisation company. The sensors were also put under a bio-burden test to check for any harmful pathogens or contaminants.

### 2.2. Instrumentation

PPG instrumentation has been developed [13,14] to drive the active elements of the sensor and process the raw signals ready for data storage on a laptop computer. The PPG device, named *ZenPPG*, has the ability to drive multiple physiological sensors and can be customised for nearly any clinical trial or in vivo study. The current configuration, chosen for these clinical trials of the endotracheal (ET) sensor, incorporates two single PPG ports, each with the capability of driving two LEDs and sensing from one photo diode. One port will be used for driving the custom ET sensor, whilst the other is designed to drive a commercial probe (Masimo, Irvine, CA, USA) for the purpose of comparison of PPGs and signal verification. This is connected to an isolation data-acquisition (DAQ) card (National Instruments, Austin, TX, USA) which is in turn connected to a laptop computer running the DAQ and data-logging software (LabVIEW, National Instruments, USA).

The LabVIEW virtual instrument (VI) developed to run ZenPPG acquires data at a rate of 1 kHz, and can control the individual brightness of individual LEDs on the attached sensors. Data is saved in the form of a text file, and includes the capability to manually time stamp the data file to aid in the synchronisation of data with events in the clinical trial, i.e., patient movement or the noting down of specific vital readings from the commercial monitors.

### 2.3. Clinical Protocol

**Figure 2:**
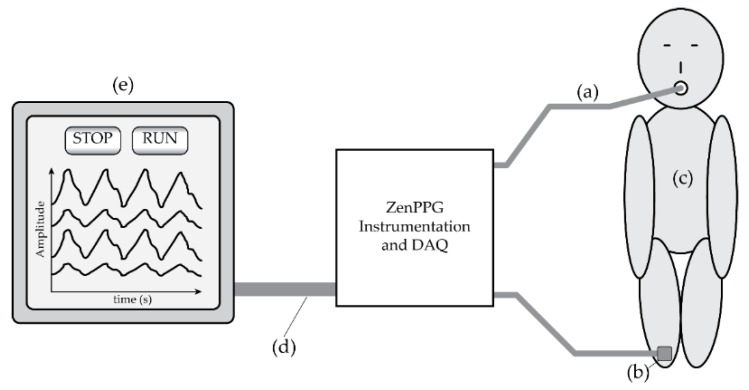
A system diagram of the tracheal PPG monitoring set-up. (**a**) The cable connecting to the ET sensor. (**b**) The reference peripheral PPG sensor and cable, which was placed on either the finger or toe of the patient, depending on where the commercial sensor was placed. (**c**) The patient. (**d**) The cable connecting the Zen instrumentation and data acquisition device to the laptop computer. (**e**) The laptop computer containing the data-logging and signal visualisation software.

The initial clinical trial was set up to establish whether or not PPG signals can be obtained from the tracheal wall, and to assess their suitability in pulse rate measurement and SpO_2_ estimation. These will be compared with the same readings as taken by the commercial monitors, as well as with a commercial sensor (Masimo, CA, USA) attached to the ZenPPG system. Figure 2 illustrates the system used for the tracheal PPG monitoring.

Following ethical approval from City London and East NHS Research Ethics Committee, London, UK, 10 patients (6 female, 4 male), median age 41.5 years (SD 16.8 years), median weight 75.5 kg (SD 19.7 kg), were recruited from surgical lists at Bart’s NHS Trust, London UK. Each patient was recruited into the study by a GCP (Good Clinical Practice) trained clinical member of the investigation team. Each recruit was given an information sheet explaining the nature of the study and adequate time to decide (48 h) if they wanted to participate. A consent form was acquired from willing recruits and after the induction of anaesthesia an endo-tracheal tube with a pre-attached ET sensor was placed into the trachea as per normal intubation procedure.

**Figure 3:**
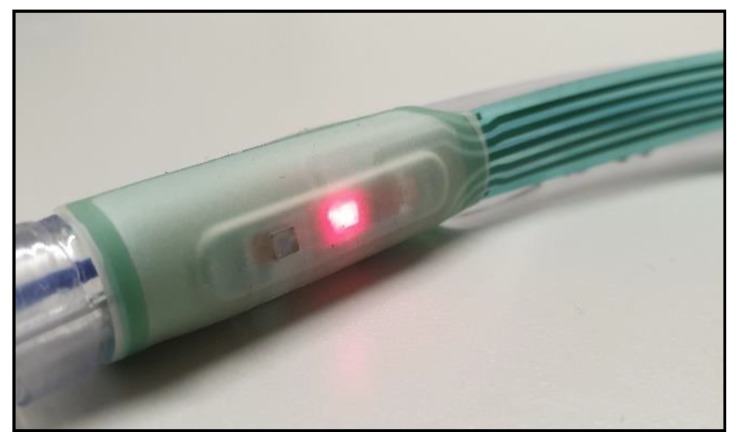
The ET Sensor attached to a standard adult size 7 ET Tube.

Prior to intubation the sensor was checked for operability (sensor attached to ZenPPG and LEDs operated by software), then adhered to the tube above the inflatable cuff just past the laryngeal guidance markers. The sensor was situated on the inside curve of the tube so that the sensor would face anteriorly. Figure 3 shows the adhesion of the sensor on the tube just below the inflatable cuff. The tube was then placed by normal intubation procedures with the sensor sitting past the larynx near the subglottis. This was visually confirmed by the Anaesthetist. The sensor was switched on once the patient was on the operating table and in a secure position.

Recordings commenced from a time when adjustments to LED intensities were made so that PPG signals from the ET sensor could be observed on the laptop display from both the infrared and red channels. Recordings were complimented with manually-entered time markers that were taken approximately every minute. Each marker was accompanied by an SpO_2_ and pulse rate reading from the clinical monitors for comparison during data analysis.

Recordings were ceased after approximately 1 h. The ET sensor was detached from the ZenPPG but left in situ for the remainder of the surgery. The sensor was removed by the anaesthetist on waking the patient as part of the normal surgical protocol.

## 3. Results

The data was analysed offline using MATLAB (The MathWorks, Natick, MA, USA). The clinical readings were used in the following comparative analyses. In addition to these clinical readings, PPG signals were recorded from a periphery (PER) location using the commercial probe already described. All time stamps were synchronised with a reading taken from the commercial monitor used by the anaesthetist (Phillips probe and processing module on a Drager Anaesthetic Machine).

### 3.1. PPG Analysis

Good quality PPG signals have been acquired from the trachea of all patients at both wavelengths. Signals were visually inspected and the longest section of uninterrupted good quality signals were selected for preliminary analysis from each patient. As can be seen in Figure 3 the signals from the ET tube were heavily modulated by ventilator artefact. Considering the location of the sensor this was expected and was easily filtered out prior to the main analysis.

The original raw PPG signals were recorded as a mixed AC + DC signal, where the AC portion is the part attributed to the volumetric pulse of the blood in the arteries. The DC portion was used to calculate the normalised PPG signal for each wavelength, and also in computing the signal to noise ratio (SNR) for each PPG wavelength from the ET sensor and the PER sensor

**Figure 4:**
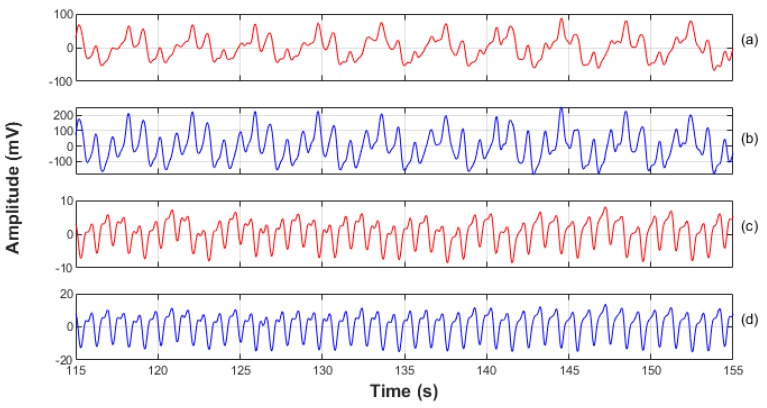
A 40 s sample of normalised PPG signals. For illustrative purposes the DC component, and high frequency noise have been filtered out. (**a**) The red PPG signal from the ET sensor. (**b**) The infrared signal from the ET sensor. (**c**) The red signal from the periphery sensor. (**d**) The infrared signal from the periphery sensor. It can be seen that all signals show a periodic modulation; this was caused by the ventilator and was removed with a high pass filter prior to signal analysis and vital-sign computations.

Figure 4 shows a typical 40 s trace of normalised PPG signals at both wavelengths from both sites, the trachea and the periphery location.

**Figure 5:**
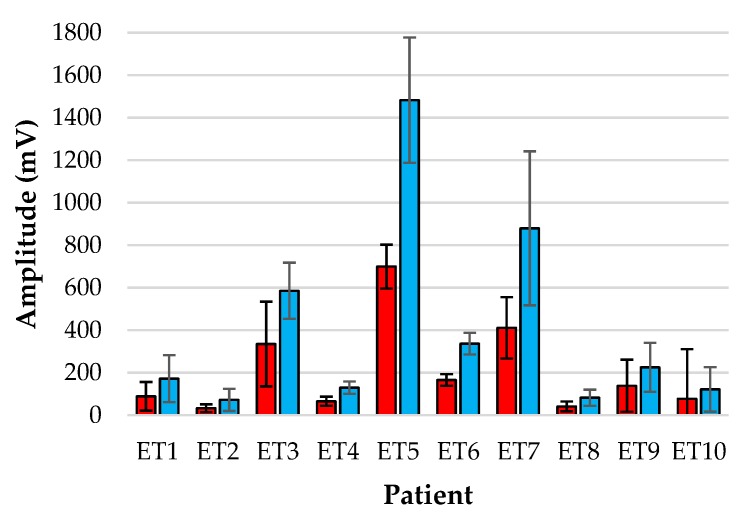
Amplitude analysis of normalised red (red) and infrared (blue) PPGs from the tracheal tube sensor for each patient over the monitoring period.

**Figure 6:**
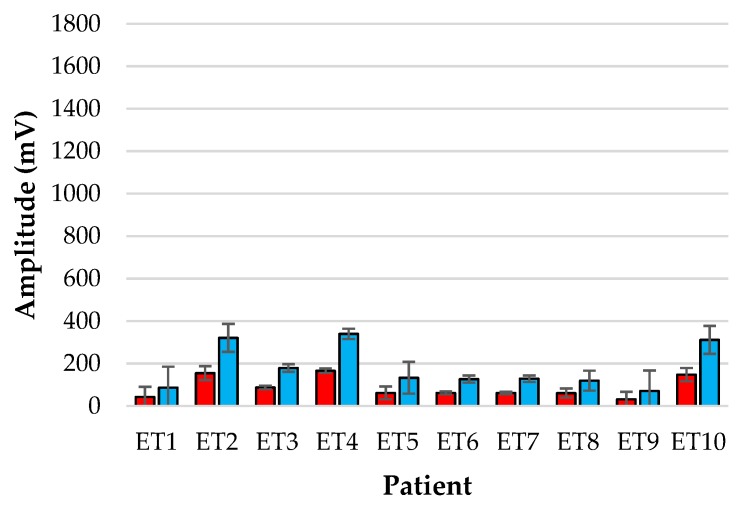
Amplitude analysis of normalised red (red) and infrared (blue) PPGs from the reference periphery sensor for each patient over the monitoring period.

Figure 5 and Figure 6 show the bar-plot of the amplitude analysis from the individual patients for the ET sensor and the periphery sensor, respectively. The large variance seen in the ET signals as opposed to the PER signals is largely due to the results from patients ET3, ET5 and ET7 who displayed much greater AC amplitudes than the other subjects. Interestingly the same patients displayed smaller normalised amplitudes at the periphery locations.

**Figure 7:**
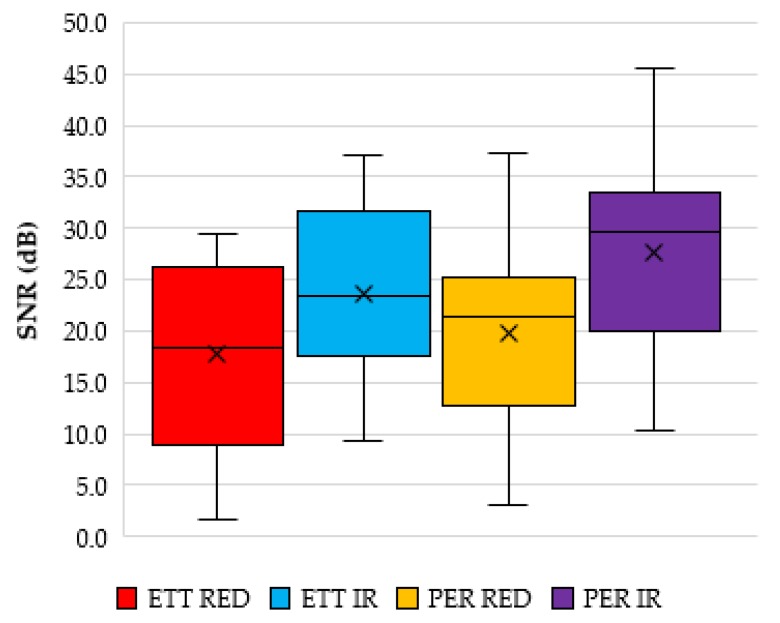
Signal to noise ratio analysis of the PPG signals from the ET and periphery (PER) sensors.

Calculating the signal to noise ratio (SNR) of the red and infrared signals reveals an average SNR value for the red and infrared signals from the ET sensor to be 17.7 dB (STD 10.1 dB) and 23.7 dB (STD 9.7 dB), respectively. The SNR from the periphery location was 19.8 dB (STD 9.8 dB) and 27.5 dB (STD 10.4 dB) for the red and infrared PPGS, respectively. Figure 7 shows a box and whisker plot of these values.

### 3.2. Vital Sign Analysis

All signals were analysed in 10-s segments at each time stamp (corresponding to a clinical reading), and passed through a custom algorithm that calculated pulse rate via a fast-Fourier-transform method and SpO_2_ using the method set out in [15] and shown in Equations (1) and (2).

(1) $$\mathrm{Sp}O_{2}=110-25R$$

where:

(2) R=ACREDDCREDACIRDCIR

Equation (1) is a generalised SpO_2_ formula where R is the ratio of the normalised red and infrared AC signal. The normalised signal is simply the ratio of the AC part of the PPG waveform to the DC waveform.

#### 3.2.1. Blood Oxygen Saturation Analysis

**Figure 8:**
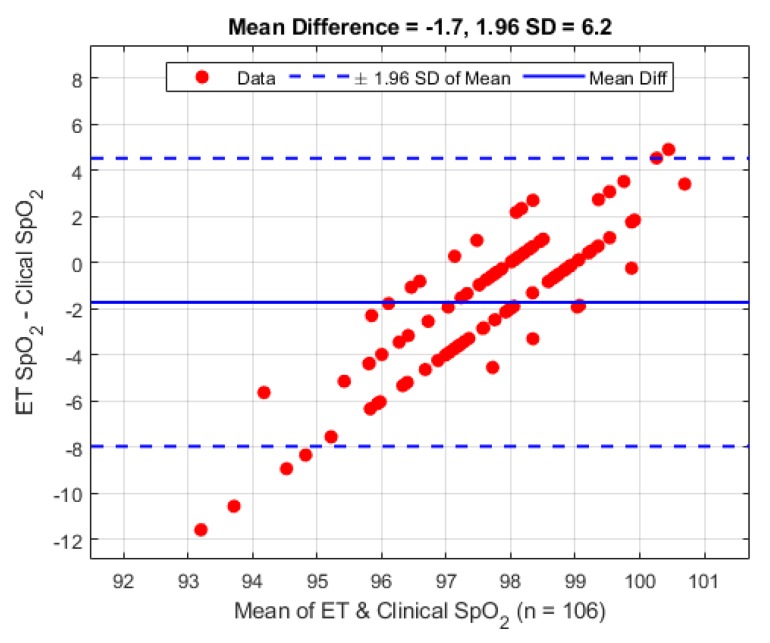
Bland–Altman analysis of SpO_2_ from the ET sensor vs. the clinical monitor.

There were a total of 106 clinical observations from the 10 patients. The SpO_2_ observations have been analysed using the Bland–Altman [16] analysis technique and are shown in Figure 8. On average the mean difference between the ET sensor and the clinical device is −1.7% (1.96 STD 6.2%).

**Figure 9:**
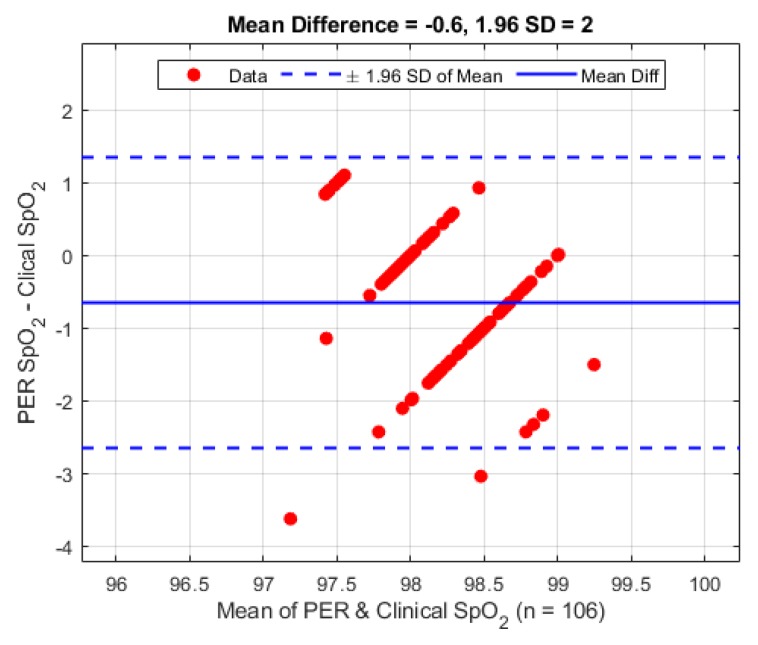
Bland–Altman analysis of SpO_2_ from the periphery sensor vs. the clinical monitor.

A second Bland–Altman analysis was carried out on the periphery sensor against the clinical data for verification purposes, and is presented in Figure 9. The periphery sensor shows a mean difference of −0.6% (1.96 STD 2.0%).

The overall mean blood oxygen saturation value calculated from the ET and PER sensor for each patient against the clinical reading is given in Table 1. The clinical measurements were taken from the pulse oximeter attached to the commercial device used by the anaesthetist.

#### 3.2.2. Pulse Rate Analysis

**Figure 10:**
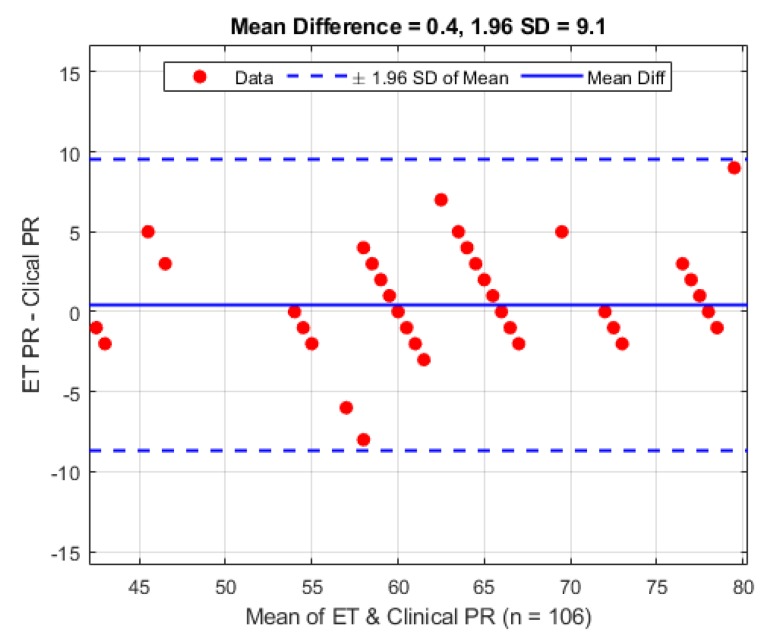
Bland–Altman analysis of pulse rate from the ET sensor vs. the clinical monitor.

The same 106 clinical observations made from the 10 patients were also made for the pulse rate and have been similarly analysed using the Bland–Altman [15] analysis technique and are shown in Figure 10. On average the mean difference between the ET sensor and the clinical device is 0.4 bpm (1.96 STD 9.1 bpm).

**Figure 11:**
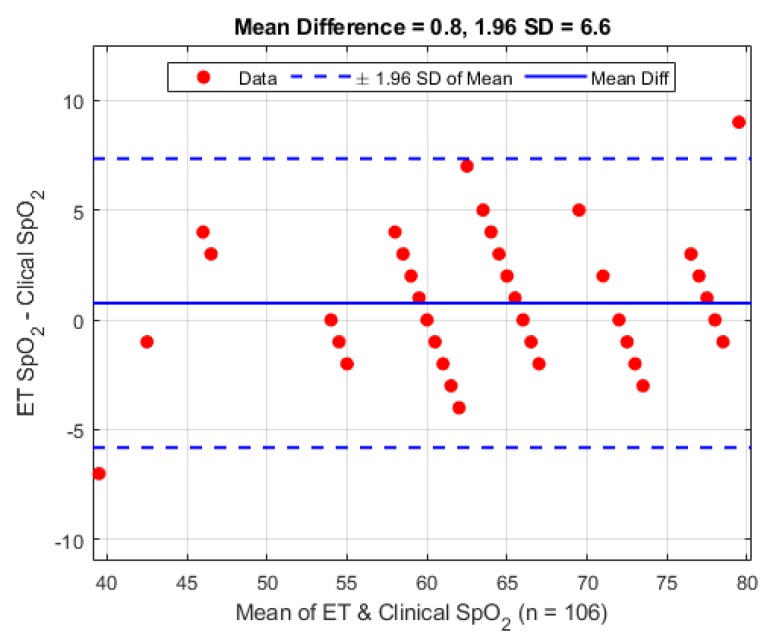
Bland–Altman analysis of pulse rate from the periphery sensor vs. the clinical monitor.

A second Bland–Altman analysis was carried out on the periphery sensor against the clinical data for verification purposes, and is presented in Figure 11. The periphery sensor shows a mean difference of 0.8 bpm (1.96 STD 6.6 bpm).

Individual results of the pulse rate calculated from the ET and PER sensor for each patient against the clinical reading is given in Table 2. Each reading is the mean pulse rate of all time stamps calculated for each patient, and was calculated from the infrared PPG signal, since they showed the highest SNR at each sensor site. The clinical measurements were taken from the pulse oximeter attached to the commercial device used by the anaesthetist.

## 4. Discussion

The study has shown for the first time the feasibility of acquiring PPGs signals from the human trachea. Initial observation of the PPG signals from the tracheal sensor compared to the reference sensor at the periphery location reveal that the trachea has overall larger normalised amplitude PPG signals compared to the periphery location. This fits with the assumption that the trachea, being at a central anatomical location, and close to the supporting network of blood vessels from the heart, would have a richer blood supply and be better perfused. However the variance of the PPG signals is greater, and the SNR lower. This may suggest that either the trachea is more susceptible to variations in blood supply caused by sympathetic responses, or that the sensor itself may not have been adequately located during the placement procedure.

As there was no way to confirm the sensor location after placement, or what the exact orientation of the tube was after the patient was moved to the operating table it is our belief that the variance issue was due to the later reasoning. It is observed that patients 3, 5, 7 and (to a lesser extent) 6 exhibited tracheal PPG amplitudes much greater than the corresponding PPG signals from the periphery location. This observation may be due to the previous assumption of the blood supply being greater and hence the tissue being better perfused or that the sensor was more adequately located. Rigorous testing would be needed to test this hypothesis. During sensor placement considerable effort was made by the anaesthetist to keep the orientation the same for every patient (sensor facing anterior) so that the same tissue was being observed in all patients. The results of the SpO_2_ calculations and corresponding comparative evaluation in Figure 8 and Figure 9 are encouraging for the future exploration of utilising the trachea to monitor SpO_2_ as an alternative to traditional locations as the overall difference is less than 2%. The larger than expected standard deviation compared to the reference sensor may easily be explained by the previously discussed item of signal amplitude variance and low SNR which would alter the ratio of ratios value needed to compute SpO_2_.

As mentioned in the methods section reflectance pulse oximeter sensors are normally constructed so that the LEDs are equidistant from the detector. Work done with fibre optics [17,18] shows optimum separation at 3 to 6 mm. Additionally there are also simulated computer models confirming that for the wavelengths of 660 nm and 940 nm in a finger model, an ideal separation distance <1 cm yields optimal depth of light penetration for SaO_2_ values >90% [19]. Components available at the time of the design of the sensor meant that a compromise had to be made in the placement and geometrical arrangement. Acquiring PPGs from the trachea was seen as a priority over whether or not this geometry was optimised for pulse oximetry, and the experiment was commenced with the compromised design.

As the physical distance of these components was greater than the suggestion made in the literature it can be reasonably concluded that the signals required for SpO_2_ calculation were not of optimum quality. The recent introduction of dedicated pulse oximeter sensors from likes of Osram Opto Semiconductors (Regensburg, Germany) (BIOFY Series) does open up new opportunities in sensor design, offering miniature one-chip pulse oximeter sensor solutions with optimised reflectance component placement, ideal for this type of sensor, and may solve the issue discussed.

Calculating pulse rate from the AC component of a PPG signal is a common and well established method of determining heart rate in patients. The pulse rate seen at the trachea varied by less than one beat compared to the periphery sensor and the commercial monitor. The standard deviation seen across individual patients shown in Table 2 was only varied due the fact that the heart rate naturally has a large range between individuals. However, the standard deviation between the three different monitoring sites has little variation, and can be considered negligible since heart rate is only ever reported in whole integer values. The Bland–Altman analysis shows a relatively high variance, but on careful inspection of the timestamps gathered in the trial there are a small number of pulse rate calculation errors where the algorithm was not able to detect the pulse rate frequency needed for pulse rate estimation. A subsequent inspection of the signals involved shows some movement artefact at some of these points and for others the signal has some high frequency interference, causing an under or overestimation in HR, respectively.

## 5. Conclusions

In this study it has been shown that good quality photoplethysmographic (PPG) signals can be measured in the human trachea. Simultaneous infrared PPG signals from identical reflectance probes in the trachea and on a finger were recorded and compared. On the basis of the data obtained, we conclude that the trachea PPG signals are of good quality with relatively good signal to noise ratio.

The close agreement between the calculated values of arterial blood oxygen saturation in the trachea and the commercial oximeter values support the hypothesis that the trachea may be an effective alternative site for monitoring blood oxygen saturation. Further studies are needed to establish whether the trachea can be used for monitoring blood oxygen saturation in patients with poor periphery circulation in whom conventional pulse oximetry fails.

## Figures and Tables

**Table 1:** The mean SpO_2_ reading from each patient for the ET, PER and clinical sensors. Standard mean, median and standard deviation of the results is shown.

Patient	ET SpO_2_ %	PER SpO_2_ %	Clinical SpO_2_ %
ET 1	96.3	97.9	98.5
ET 2	97.9	98.0	98.7
ET 3	95.4	97.8	97.3
ET 4	97.4	97.8	98.0
ET 5	97.9	98.1	100.0
ET 6	97.6	97.8	98.4
ET 7	97.1	98.1	99.0
ET 8	97.7	96.9	98.1
ET 9	95.4	97.9	99.0
ET 10	97.8	98.2	99.0
**MEAN**	**97.1**	**97.8**	**98.6**
**MEDIAN**	**97.5**	**97.9**	**98.6**
**STD**	**1.0**	**0.4**	**0.7**

**Table 2:** The mean pulse rate reading from each patient for the ET, PER and Clinical sensors. Standard mean, median and standard deviation of the results is shown.

Patient	ET PR (bpm)	PER PR (bpm)	Clinical PR (BPM)
ET 1	62.8	64.0	63.0
ET 2	44.1	44.4	43.4
ET 3	65.6	65.7	64.2
ET 4	66.0	66.0	65.3
ET 5	60.0	60.0	60.4
ET 6	77.9	78.0	76.5
ET 7	76.4	76.4	74.7
ET 8	68.4	69.0	69.3
ET 9	73.8	69.6	73.7
ET 10	58.6	58.6	56.9
**MEAN**	**65.4**	**65.2**	**64.7**
**MEDIAN**	**65.8**	**65.9**	**64.7**
**STD**	**10.0**	**9.6**	**9.9**
